# Countering Coloniality in Global Health: Comment on "The Rhetoric of Decolonizing Global Health Fails to Address the Reality of Settler Colonialism: Gaza as a Case in Point"

**DOI:** 10.34172/ijhpm.8670

**Published:** 2024-09-23

**Authors:** Autumn Asher BlackDeer

**Affiliations:** Graduate School of Social Work, University of Denver, Denver, CO, USA.

**Keywords:** Decolonization, Colonialism, Coloniality, Global Health

## Abstract

This commentary joins the chorus of rightful critiques of global health as it continues to further colonial agendas under the guise of supposed well-meaning efforts. Engebretsen and Baker rightfully call out the uptake of decolonial rhetoric in the field of global health, pointing out notable failures to actually challenge undergirding colonial structures and move beyond theory into meaningful action, using clear examples from the ongoing crisis in Gaza and global health’s ongoing response (or lack thereof). In this work I bring together essential foundations of decolonial scholarship in order to further the work Engebretsen and Baker have defined as crucial reckoning points for the field of global health. This commentary will (1) ground our conversation by defining true decolonization, (2) delineate the coloniality of knowledge and its manifestations in global health, and (3) conclude with a call to develop a decolonial praxis.

## Countering Coloniality in Global Health

 Several have illuminated patterns of colonization throughout the history of global health, from ignoring structural determinants of health such as slavery, environmental racism, and predatory capitalism; to the coloniality of knowledge such as racial inferiority science, privileging of Euro-Western knowledge systems, and the centering of Global North perspectives as universally beneficial; to the blatant acts of colonialism perpetuated under the guise of medical progress, such as the Tuskegee Experiment, the Guatemala Syphilis Study, the epidemic of forced sterilization of marginalized bodies, and the restriction of movement of Indigenous peoples for purported infection control.^1,2^ Thus, to counter this coloniality in global health, decolonization is imperative. At its most fundamental level, decolonization is the undoing of colonialism that brings about the repatriation of Indigenous land and life^3^ – this can look like a myriad of practices and ways of being in the world. For some, decolonizing is reclaiming traditional ways of knowing and being; for others, it is working to exorcise the inner colonizer within our minds. The logical endpoint of decolonization is dismantling harmful structures that perpetuate colonialism in the present day. Thus, to meaningfully decolonize, we must first name and identify the root cause of these ongoing issues: colonialism. Colonization is a global phenomenon whereby colonizers force their agendas onto Indigenous populations for land, power, and ultimate control. Decolonization is inherently political as it upholds that Indigenous people deserve sovereignty and self-determination over their lands and livelihoods. Engebretsen and Baker^4^ clearly call out the World Health Organization’s (WHO’s) stance on the ongoing assault of occupied Palestinian territories and how the WHO continues to overlook these connections to settler colonialism as they fail to address their entanglement with colonial logics and processes. This global health organization is complicit in colonial apologist approaches at best – putting out passive tense statements on the attacks in Gaza which fail to explicitly name the aggressor, to blatantly furthering Israel’s genocidal agenda at worst – leaving their colonial power unchallenged, waiting to address the aftermath as a humanitarian crisis once Israel has completed its Palestinian genocide.^4^

 In order to meaningfully engage with decolonial work, we must first cut through all the noise and jargon that have become associated with decolonization. As Engebretsen and Baker^4^ identify as decolonial rhetoric, the general population certainly understands decolonization as another form of the DEI enterprise: diversity, equity, and inclusion, as it has been superficially absorbed as another way of talking about social justice.^3^ Decolonization comes from an entirely different framework; whereas DEI centers on social justice, reforming systems, diversifying them and making them more inclusive under their twisted version of equity, decolonization is about undoing these systems altogether. To that end, decolonization is aligned with the larger movement for abolition, which also holds that reforms will never work because the system is functioning exactly how it was designed to. Reforms to these systems or removing bad actors will never result in the necessary changes because the system itself will continue to churn out its colonial agenda regardless. This can be seen in the very nature of global health as a concept.

 Global health itself is a ubiquitous concept; rather than a specific, place-based practice or movement, global health uses the illusion of an international focus while still combing over colonialism globally; institutions of global health in the Global North perpetuate Eurocentric worldviews that fail to consider the majority of the world’s actual needs.^1^ Both Engebretsen and Baker^4^ and Indigenous scholars Jensen and Lopez-Carmen^5^ have identified the ways in which global health has positioned itself to be mostly concerned with low- and middle-income countries (LMICs) outside their borders, typically in the global south. Further, Jensen and Lopez-Carmen^5^ note how they have been silenced in decolonizing global health discussions, with white US physicians pushing them to make a separate paper on “Indigenous issues.” This brings to bear the elephant in the room, that Native Nations and settler colonialism are rarely considered within global health, even in decolonizing global health spaces. I would argue that global health hides behind this lack of place-based specificity, never having to name or claim any legacies of colonial harm. This is reflected in what Engebretsen and Baker^4^ illuminate, that the field of global health stands idly by wringing their hands in the aftermath of colonial violence, preferring to wait and then respond later as a humanitarian crisis within which they have no blood on their hands. These are fundamental “settler moves to innocence” as coined by Tuck and Yang^3^ in their seminal work, Decolonization is Not a Metaphor. Ultimately, this ubiquitous nature of global health allows it to be everywhere and nowhere at the same time, accountable to no one community, nation, or population. This ubiquity also allows global health to be taken up by well-meaning white folks who want to save the world; except they’re only interested in saving certain populations that fit within their worldview of whiteness, which Israel certainly does.^6^

## Coloniality of Knowledge

 Throughout Engebretsen and Baker^4^ work are tremendous examples of the ways in which global health perpetuates colonialism through the coloniality of knowledge. Put forward to encapsulate the ongoing nature of colonialism beyond the colonial time period, coloniality is described as a conceptual apparatus, transcending racial, political, economic, social, and epistemological hierarchies imposed by European colonization.^7^ Ultimately, coloniality refers to these long-term patterns of power that we see across culture, labor, and knowledge production.^7^ There is a broader colonial matrix of power which encapsulates domains of the coloniality of: being, gender, nature, and knowledge^7^; however, for the purposes of brevity, we will only consider the coloniality of knowledge in global health here.

 Engebretsen and Baker describe a top-down knowledge production process in global health, a sentiment echoed in Hussain and colleagues’^2^ in-depth delineation of colonization and decolonization of global health. The history of global health has established the Global North as a leader in the field,^1^ defining high-income countries as the leaders of the field and thereby setting the global standard; however, Jensen and Lopez-Carmen^5^ demonstrate how the United States claims leadership on a global scale yet most Native Nations located within the United States are virtually invisible. This hierarchical presentation of knowledge is one of the foundations of coloniality; just as Engebretsen and Baker illuminate about global health, these hierarchies are self-insulating, ensuring that those on top stay on top and continue to control the narrative, as evidenced through the “suppression of local insights, notably from regions such as Palestine” (p. 2).

 In addition to hierarchical dominance, the coloniality of knowledge is further defined by the concept of distance. Māori scholar Tuhiwai Smith illuminates the problem of positivism as a key defining factor in white western colonial logic in her seminal work on Decolonizing Methodologies. In her first chapter on Research through Imperial Eyes, she describes how understanding the world through measurement flattens our understanding to focus on issues of procedure and operationalized definitions rather than true depth of a subject.^8^ These issues of distance in global health’s coloniality of knowledge are identified by Engebretsen and Baker through the field’s ongoing use of the passive voice, waiting for the aftermath of colonial violence to then lament over the tragedies. Hussain and colleagues^2^ similarly delineated the perpetuation of colonial relationships in global health as members of high-income countries are given greater opportunities within LMICs, rather than the other way around. This pattern leads to high income global health participants to travel to LMICs to save these communities,^2^ or bring progress – common colonizing party lines. These same sentiments have surrounded the genocide in Gaza, stating that Israel is on a colonizing mission to eradicate the “human animals”^[1]^ of Palestine.^9^ Where is the outrage from global health over this blatant dehumanization? How can global health claim any role in decolonizing while allowing this genocide to continue?

## Towards a Decolonial Praxis

 As several have called out, global health must move beyond the rhetoric of decolonization and towards meaningful action and embodiment. While some have suggested those from high-income countries should start questioning their role in global health altogether, they caution against violence and conclude by acknowledging biases^2^ which again, falls just short of charting a path towards decolonization. While global health should certainly name and recognize settler colonialism and how it is contributing to its perpetuation, acknowledgement is not enough. Decolonial praxis is best described by the 1996 quote by Paulo Freire,^10^ “Discovery cannot be purely intellectual but must involve action; nor can it be limited to mere activism but must include serious reflection: only then will it be a praxis” (p. 26). Global health must move from the theoretical rhetoric as outlined by Engebretsen and Baker^4^ and towards meaningful, informed action. In order to begin dismantling the coloniality of knowledge in global health, it is important to begin decentering hierarchies and centering those most impacted by colonialism. Engebretsen and Baker^4^ are clear in their call for decolonial work clearly defined and realized through direct engagement with those most impacted by colonialism and defining the path forward on their terms. As for the current case in Gaza, it is clear that decolonization must center Palestinians and how they define liberation. As I always say, we have all been impacted by colonialism, thus it is the responsibility of us all to decolonize. The question remains, will global health rise to the occasion or continue to hide behind its appearance of benevolence?

## Ethical issues

 Not applicable.

## Conflicts of interest

 Author declares that she has no conflicts of interest.

## Endnote


^[1]^ Excerpt from quote by Israeli Defense Minister Yoav Gallant while announcing complete siege of the Gaza Strip.^9^
